# PDK1 is a negative regulator of axon regeneration

**DOI:** 10.1186/s13041-021-00748-z

**Published:** 2021-02-12

**Authors:** Hyemin Kim, Jinyoung Lee, Yongcheol Cho

**Affiliations:** Department of Life Sciences, Korea University, Anam-ro 145, Seongbuk-gu, Seoul, 02841 Republic of Korea

**Keywords:** PDPK1, PDK1, PNS, CNS, Axon regeneration, DLK, Kinase

## Abstract

Axon regeneration in the central nervous system is inefficient. However, the neurons in the peripheral nervous system display robust regeneration after injury, indicating that axonal regeneration is differentially controlled under various conditions. To identify those molecules regulating axon regeneration, comparative analysis from dorsal root ganglion neurons at embryonic or adult stages is utilized, which reveals that PDK1 is functions as a negative regulator of axon regeneration. PDK1 is downregulated in embryonic neurons after axotomy. In contrast, sciatic nerve axotomy upregulated PDK1 at protein levels from adult mice. The knockdown of PDK1 or the chemical inhibition of PDK1 promotes axon regeneration in vitro and in vivo. Here we present PDK1 as a new player to negatively regulate axon regeneration and as a potential target in the development of therapeutic applications.

## Introduction

Although most neurons fail to regenerate axons in the central nervous system (CNS), some neurons in the peripheral nervous system (PNS) successfully regenerate their injured axons to fully recover their original function [1]. The robust axon regeneration in the PNS is orchestrated by multiple kinases responsible for regulating injury responses [2–6]. Therefore, identifying the kinases that regulate regeneration offers the opportunity to manipulate the intrinsic regenerative potential by directly modulating kinase activity and related pathways.

Phosphoinositide 3-dependent kinases are involved in regulating many biological processes [7]. For example, 3-phosphoinositide-dependent protein kinase-1 (PDK1), regulated by growth factors and hormones, is a pivotal member of this signaling pathway. PDK1 has been found to play an essential role in cell survival, differentiation, and proliferation via the phosphorylation and activation of the AGC protein kinase family, including protein kinase B (PKB/AKT), p70 ribosomal S6 kinase (S6K), serum- and glucocorticoid-induced protein kinase, and protein kinase C (PKC) [7]. In addition, PDK1 is an important target to understand cancer development [8]. In neurons, PDK1 is known to have a role in proliferation, migration, and neurogenesis in the developmental stage [9] and the progression of neurodegenerative diseases such as Alzheimer’s and prion disease [10]. However, to date, no research has been conducted on the function of PDK1 in axon regeneration. In the present study, we present PDK1 as a negative regulator of axon regeneration. PDK1 is found to be differentially regulated at the protein level in response to axonal injury, depending on the developmental stage of the sensory neurons. The knockdown or chemical inhibition of PDK1 significantly upregulates axon regenerative potential in vitro and in vivo. PDK1 is involved in DLK/c-jun pathway because the protein levels of DLK and c-jun are downregulated when PDK1 is knocked down. PDK1 is thus a potential target for the development of therapeutic applications for axon regeneration.

## Results

### Axotomy induced the downregulation of axonal PDK1 in embryonic DRG neurons in vitro

**Fig. 1:**
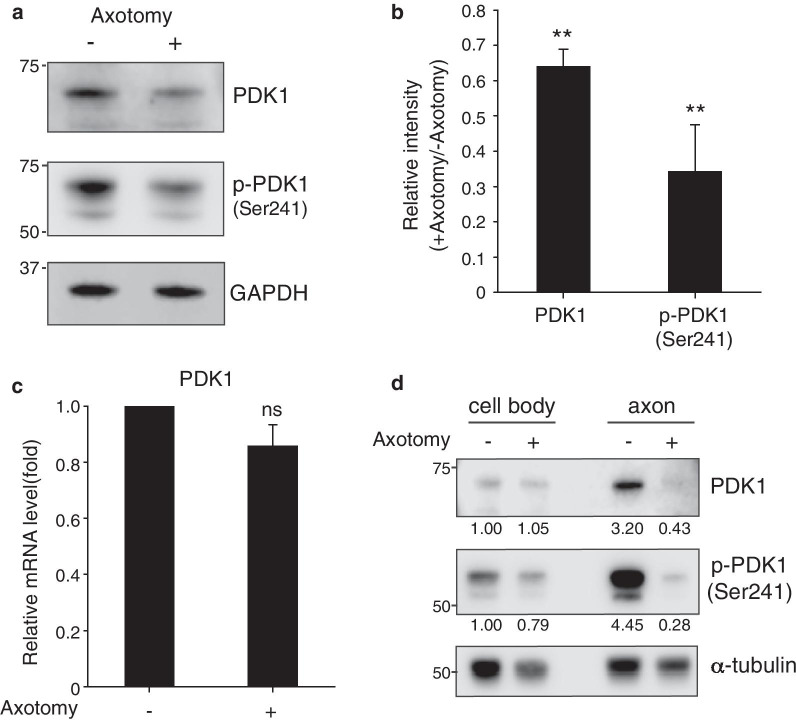
PDK1 is downregulated in vitro after axonal injury in cultured embryonic DRG neurons. **a** Western blot analysis of embryonic DRG neuron cultures with (+) or without (−) axotomy. Protein lysates were prepared 6 h after axotomy. p-PDK1 (Ser241) is PDK1 phosphorylated at serine 241. **b** Relative intensity of PDK1 and p-PDK1 (Ser241) Western bands from (**a**) (Mean ± S.E.M.; n = 3, **p < 0.01 from a *t*-test). **c** Relative mRNA levels measured using quantitative RT-PCR from cultured embryonic DRG neurons with (+) or without (−) axotomy (n = 3; ns from a *t*-test). **d** Western blot analysis of protein lysates from the cell bodies and axons prepared separately with (+) or without (−) axotomy. Numbers indicate normalized relative intensities

To understand the role of PDK1 in axon regeneration, we utilized in vitro sensory neuron cultures prepared from embryonic mouse dorsal root ganglions (DRGs) because of their high regenerative capacity [11] and found that axotomy induced downregulation of PDK1 at the protein level (Fig. 1a). Analysis of the total protein lysates prepared from mouse embryonic DRG neurons revealed that axotomy reduced PDK1 protein levels without significantly changing the mRNA levels (Fig. 1b and c), suggesting that PDK1 downregulation is regulated at the protein level after injury. Because axonal injury leads to signal transduction both locally at the injury site and distantly in the cell bodies, protein lysates were prepared separately from the axons and the cell bodies and subjected to Western blot analysis using a previously reported spot-culture system [12–14]. It was found that PDK1 is highly concentrated in the axons of uninjured embryonic DRG neurons and that axonal PDK1 levels were lower following injury (Fig. 1d). Overall, these results indicate that axonal injury induces the downregulation of axonal PDK1 at the protein level in embryonic DRG neurons in vitro.

### PDK1 protein levels were upregulated in sciatic nerves after injury

**Fig. 2:**
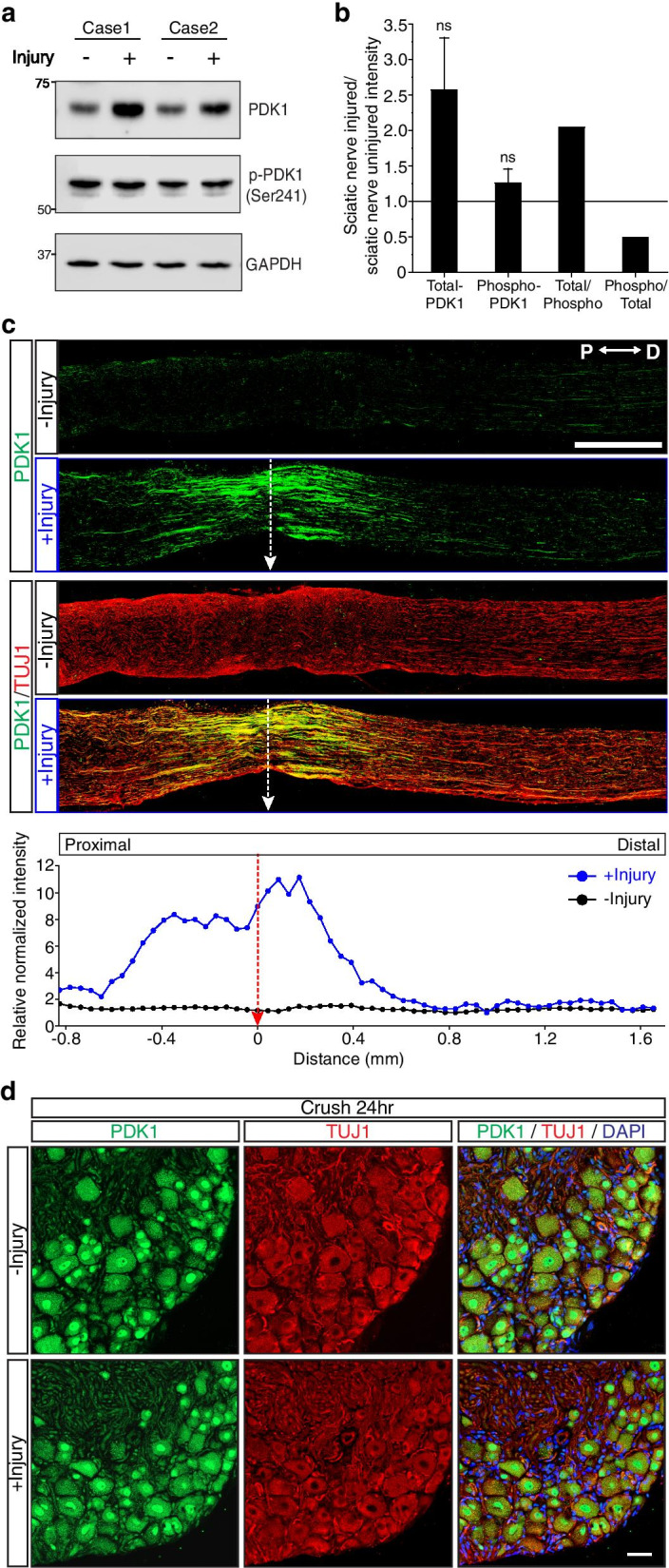
PDK1 is upregulated in vivo at sites adjacent to the injury in mouse sciatic nerves. **a** Western blot analysis of protein lysates from mouse sciatic nerves with (+) or without (−) injury. Protein lysates were prepared 24 h after axotomy. **b** Relative intensity of PDK1 and p-PDK1 (Ser241) Western bands from (**a**) (Mean ± S.E.M.; n = 5, ns from *t*-test). **c** Representative immunohistochemistry of longitudinal sections of mouse sciatic nerves with (+) or without (−) injury. P, proximal to the DRG tissues, D, distal to the nerve terminus. Scale bar, 500 μm. Relative normalized intensity of PDK1 is plotted with x-axis of distance and y-axis of relative ratio. **d** Representative immunohistochemistry of mouse L4 DRG tissue with (+) or without (−) injury. Scale bar, 50 μm

We then determined whether the results from embryonic DRG neurons in vitro were reproduced in adult mice in vivo. Sciatic nerves from 8-week-old mice were injured and its protein lysates were subjected to Western blot analysis. The results showed that PDK1 protein levels were upregulated by injury without any significant changes in phospho-PDK1 levels (Fig. 2a). In particular, the sciatic nerve injury increased PDK1 protein levels 2.5-fold, although this was not statistically significant (Student’s *t*-test, p = 0.07; Fig. 2b). The upregulation of PDK1 was a direct result of the injury to the axons in the sciatic nerves because longitudinal sections of the crushed sciatic nerves revealed that PDK1 immunoreactivity was highly co-localized with neuronal marker TUJ1 (Fig. 2c). In addition, immunohistochemistry analysis also found that PDK1 was specifically upregulated in areas adjacent to the injury sites (Fig. 2c). However, PDK1 levels in the neuronal cell bodies of DRG tissue exhibited no significant changes, indicating that PDK1 protein is upregulated at the site of injury in axons in vivo (Fig. 2d). Taken together, these results indicate that PDK1 protein levels are differently regulated in response to axonal injury depending on the developmental stage of the sensory neurons.

### PDK1 is a negative regulator of axon regeneration

**Fig. 3:**
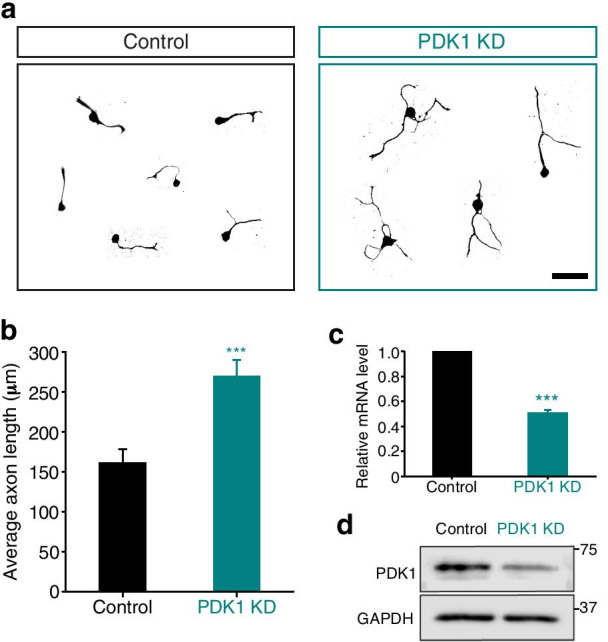
PDK1 knockdown promotes axon regeneration in vitro. **a** In vitro axon regeneration assays. Mouse embryonic DRG neurons were re-plated, fixed, and immunostained with TUJ1 antibody. Control, shRNA-control lentivirus and PDK1 KD, shRNA to PDK1 lentivirus. Scale bar, 100 μm. **b** Average length of the regenerating axons from (**a**) (Mean ± S.E.M.; n = 331 and 374 cells for each condition; ***p < 0.001 from a *t*-test). **c** Relative mRNA levels measured using quantitative RT-PCR from cultured embryonic DRG neurons to confirm the successful knockdown of PDK1 (Mean ± S.E.M.; n = 3; ***p < 0.001 by *t*-test). **d** Western blot analysis of protein lysates from cultured embryonic DRG neurons from shControl or PDK1 KD samples

Comparative gene expression profiling has revealed that the expression of regeneration-associated genes is differentially regulated in embryonic and adult DRG neurons [15, 16]. This suggests that injury-related signal transductions may differ between these developmental stages. Because the present study found that PDK1 was downregulated after axotomy in mouse embryonic DRG neurons (Fig. 1a and b), we investigated whether the lower expression of PDK1 promoted axon regeneration under the same conditions. We first knocked down PDK1 via the delivery of lentiviral shRNA and analyzed the regenerative potential of embryonic DRG neurons in vitro using re-plating assay [17–19]. The result showed that PDK1 knockdown significantly enhanced axon regeneration (Fig. 3a). The average length of regenerating axons in the control neurons was 161.3 ± 17.1 μm, whereas that in PDK1-knockdown neurons was 269.4 ± 20.9 μm, a 1.7-fold increase (Fig. 3b). The successful knockdown of PDK1 was confirmed at the mRNA and protein level by RT-qPCR and Western blot analysis, respectively (Fig. 3c and d). These results show that the knockdown of PDK1 promotes axon regeneration, suggesting that PDK1 is a negative regulator of axonal outgrowth and regeneration. This implies that PDK1 is a potential target for improving the regenerative ability of sensory neurons.

### PDK1 inhibition promotes axon regeneration in vitro

**Fig. 4:**
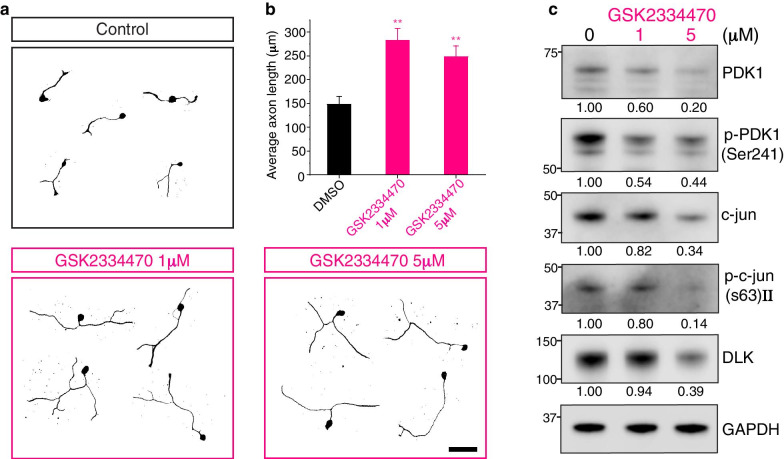
GSK2334470, a chemical inhibitor of PDK1, promotes axon regeneration in vitro. **a** In vitro axon regeneration assays. Mouse embryonic DRG neurons were re-plated in GSK2334470 (1 μM or 5 μM as indicated) or a vehicle (control) containing culture medium, fixed, and immunostained with TUJ1 antibody. Scale bar, 100 μm. **b** Average length of the regenerating axons from (**a**) (Mean ± S.E.M.; n = 151, 156, and 161 cells for each condition; **p < 0.01 from a *t*-test). **c** Western blot analysis of protein lysates from control (0, vehicle only) or GSK2334470-treated embryonic DRG neuron cultures. Numbers indicate the relative intensities

PDK1 knockdown promoted axon regeneration in mouse embryonic DRG neurons in vitro (Figs. 1a and 3a). Because PDK1 was significantly upregulated at the protein level following sciatic nerve injury in vivo (Fig. 2a and b), we hypothesized that the failure to lower PDK1 protein levels in vivo leads to weaker regenerative potential in adult DRG neurons. To test whether PDK1-mediated pathways negatively regulate axon regeneration, PDK1 inhibitor GSK2334470 was added to embryonic DRG cultures and analyzed using in vitro regeneration assays. GSK2334470 is a highly selective PDK1 inhibitor that does not affect other closely related AGC kinases [20–22]. We found that GSK2334470 dramatically enhanced the axon regeneration of embryonic DRG neurons in vitro (Fig. 4a and b). PDK1 is able to auto-phosphorylate at serine 241, which is required for its kinase activity to substrates [20, 23]. Western blot analysis showed that GSK2334470 downregulated p-PDK1 (Ser241) as well as the total PDK1 protein levels (Fig. 4c). It is known that PDK1 auto-phosphorylation leads to self-stabilization, activating downstream signal pathways via its structural changes and its phosphorylation-dependent protein interactions [24–26]. Thus, it is likely that the GSK2334470-dependent inhibition of Ser241-phosphorylation leads to PDK1 destabilization and reduces its protein levels in embryonic DRG cultures (Fig. 4c). Notably, GSK2334470 also downregulated the DLK/c-jun pathways (Fig. 4c). DLK is known to regulate injury-responsive signal transduction by modulating the JNK/c-jun pathway, thus regulating regeneration and degeneration [27]. However, there are no known upstream kinases regulating DLK pathways. Therefore, the molecular interaction between PDK1 and DLK needs to be investigated to determine whether PDK1 has a role as an upstream regulator of DLK and its downstream pathways. In addition, the molecular dynamics of GSK2334470-mediated PDK1 and p-PDK1 degradation need to be further investigated because a higher dose of GSK2334470 with 5 μM resulted in elevation of relative levels of p-PDK1/PDK1 comparing to a lower dose with 1 μM (Fig. 4c). This also implies that PDK1 and p-PDK1 might have specific roles in axon regeneration with distinct targets, which needs to be further investigated.

### In vivo GSK2334470 treatment promotes axon regeneration in sciatic nerves

**Fig. 5:**
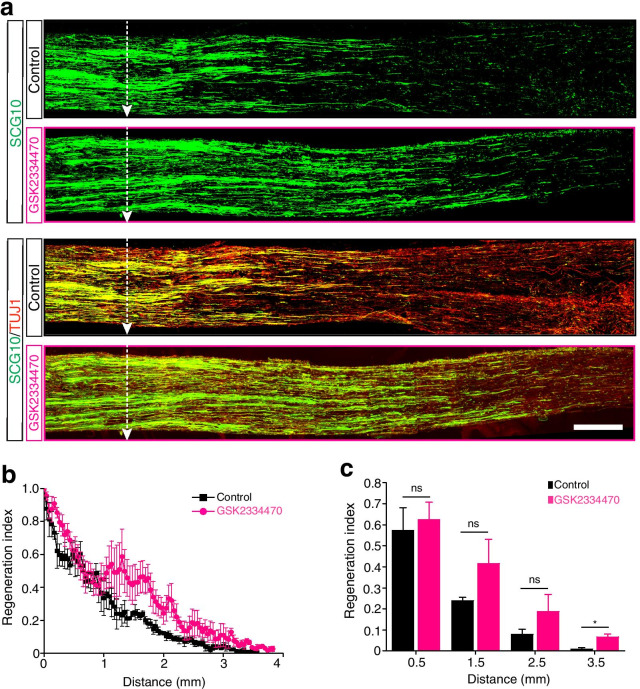
GSK2334470 promotes axon regeneration in mouse sciatic nerves in vivo. **a** Representative images of longitudinal sections of control mouse sciatic nerves or those treated with GSK2334470 (white dotted arrows represent the site of the injury). The sections are immunostained with anti-SCG10 and TUJ1 antibody. Scale bar, 500 μm. **b** and **c** In vivo regeneration index measured using ImageJ (Mean ± S.E.M.; n = 3 for each condition; *p < 0.05 from a *t*-test; ns, not significant)

Because GSK2334470 inhibited PDK1 kinase activity and reduced PDK1 protein levels in vitro, we utilized GSK2334470 to block the upregulation of PDK1 at sites adjacent to injury in mouse sciatic nerve models and test whether GSK2334470 enhances the regenerative potential in vivo. To deliver GSK2334470 to the injury site, a gel in which GSK2334470 had been dissolved was locally applied to the sciatic nerves immediately after the injury [12, 13, 28]. We found that GSK2334470 significantly promoted axon regeneration in mouse sciatic nerves (Fig. 5a). The immunostaining of SCG10, which specifically labels regenerating axons in the PNS, revealed that GSK2334470 promoted axon regeneration three days after injury compared to the vehicle-treated control (Fig. 5b and c) [29, 30]. The distal part of the injured sciatic nerves exhibited significant levels of SCG10-positive immunoreactivity, with a regeneration index that was 7.8-fold higher than the control (p < 0.05; Fig. 5c). This indicates that PDK1 is a negative regulator of axon regeneration and is a genetic and/or pharmacologic target to promote axon regenerative potential within the PNS.

### PDK1 knockdown downregulates the DLK/c-jun MAPK pathway

**Fig. 6:**
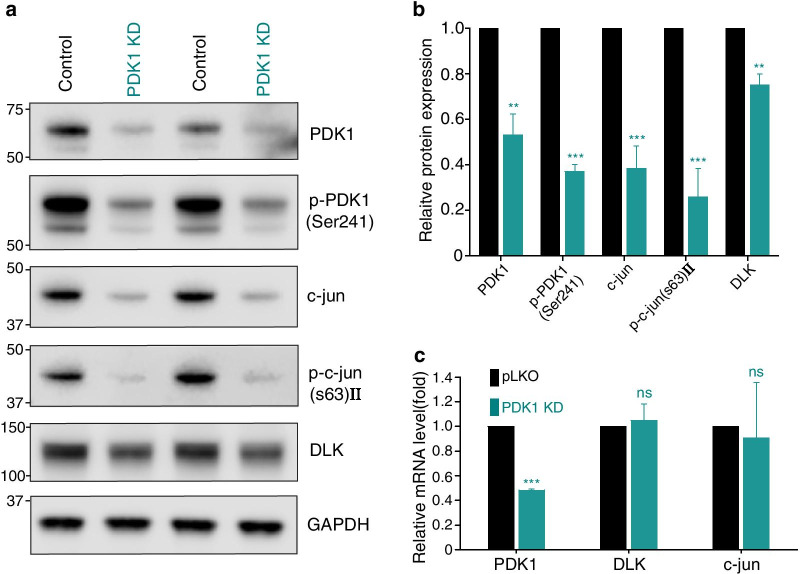
PDK1 knockdown leads to downregulation of the DLK/c-jun pathway. **a** Western blot analysis of protein lysates from control or PDK1-knockdown embryonic DRG neurons. **b** Relative intensity of (**a**) (Mean ± S.E.M.; n = 4 for each condition; ***p < 0.001, **p < 0.01 from a *t*-test). **c** Relative mRNA levels measured using quantitative RT-PCR from cultured embryonic DRG neurons (Mean ± S.D.; n = 6; ***p < 0.001 from a *t*-test)

To understand the signaling pathways involved in PDK1-mediated axon regeneration, the MAPK pathway for DLK and c-jun was investigated because DLK and its downstream effector c-jun are known to control injury-related signaling pathways of both regeneration and degeneration [27, 31–34]. We found that PDK1 knockdown reduced the protein levels of DLK and c-jun (Fig. 6a–c). The downregulation was not from transcriptional regulation because their mRNA levels did not exhibit any significant changes (Fig. 6c). These results indicate that PDK1 potentially negatively regulates axon regeneration in embryonic sensory neurons and might be responsible for modulating the DLK/c-jun MAPK pathway in mouse embryonic DRG neurons, although biochemical and genetic analysis needs to be investigated.

## Discussion

Multiple kinases have been identified as important factors regulating axonal injury-associated pathways. Among them, the DLK and JNK/c-jun are well-known kinases that regulate neuronal injury-responsive signal transduction [2, 3, 32, 35–38]. The inhibition of DLK by a small molecule inhibitor has been found to be neuroprotective under a range of conditions [33, 39–41]. This suggests that DLK is a potential target for the manipulation of the injury response of neurons. However, to date, no molecule has been identified as a potential upstream kinase of DLK from axon regeneration context. In the present study, we identified PDK1 as a negative regulator of axon regeneration in vitro and in vivo. The protein levels of PDK1 were regulated differently by axonal injury in embryonic DRG neurons and adult mice, with no significant changes in its mRNA levels at either developmental stage. Axotomy downregulated PDK1 protein levels in the axons of embryonic neurons, while mouse PDK1 protein levels were dramatically upregulated at the site of injury in sciatic nerves from adult mice. The knockdown of PDK1 and treatment with a chemical inhibitor of PDK1 significantly promoted axon regeneration in an in vitro embryonic culture model. Finally, in vivo application of the PDK1 inhibitor enhanced the regenerative potential of mouse sciatic nerves in vivo. Both PDK1 knockdown and PDK1 inhibition downregulated the DLK/c-jun pathway, which is an important regulator of the neuronal injury response, although more research is required to understand the underlying mechanisms.

The fate of neurons after injury is determined by injury-induced signal transduction [3]. Thus, the identification of the molecules involved in this process is important in developing therapeutic applications to improve the regenerative potential and thus prevent neurodegeneration and neuronal loss. We propose PDK1 as a kinase that has a negative function in axon regeneration. PDK1 is known to regulate multiple downstream kinases, including AKT [9], PKC [42], and S6K [43, 44], that are known to be important for regulating axon regeneration and degeneration. The possible function of PDK1 in axon regeneration may be involved in regulation of S6K as mTOR substrate S6K1 has been identified as a negative regulator of axon regeneration [45–47]. Because PDK1 is able to phosphorylate S6K [43], PDK1-S6K pathway may be a major signal transduction pathway negatively regulating axonal re-growth after injury. As we show that PDK1 knockdown and PDK1 inhibition promoted axon regeneration, the downstream pathways regulated by PDK1 following axonal injury need to be identified to more clearly understand the mechanisms involved and to develop PDK1-associated treatments that can enhance axon regeneration.

## Methods

### Antibodies and Western blot analysis

The following antibodies were used in the present study: anti-DLK antibody (Thermo, PA5-32173), anti-phospho-PDK1(Ser241) antibody (Cell Signaling, C49H2), anti-PDK1 antibody (Abcam, ab186870), anti-phospho-c-Jun(Ser63)II antibody (Cell Signaling, #9261S), anti-c-Jun(60A8) antibody (Cell Signaling, #9165S), anti-GAPDH antibody (Santa Cruz Biotechnology, sc-32233, clone 6C5), anti-βIII tubulin antibody (Abcam, ab41489), and anti-SCG10 antibody (Novus Biologicals, NBP1-49461).

Embryonic DRG cells were collected and lysed in 1X SDS lysis buffer at DIV5. Adult mouse tissue was dissected and lysed in RIPA lysis buffer. The supernatant of the protein lysates was collected using centrifugation. Quantified protein lysate samples were subjected to SDS-PAGE and Western blot analysis using nitrocellulose membranes that had been blocked in 5% skim milk in TBS-T for 1 h at room temperature. The bound antibodies were detected using an ECL system.

### In vivo axon regeneration assay

All procedures were approved by the Korea University Institutional Animal Care & Use Committee. All surgical procedures were performed under isoflurane anesthesia as previously described, and the sciatic nerve injury model was utilized as previously reported [48]. For in vivo axon regeneration assay, mouse sciatic nerves were crushed with forceps as previously described [12, 13, 28, 48]. The nerves were dissected at three days after injury and fixed for 1 h in 4% paraformaldehyde (Biosesang, P2031), and left overnight in PBS with 30% sucrose. The tissue embedded in OCT was sectioned and stained with the indicated antibodies. To assess axon regeneration, SCG10 fluorescence intensity was measured as previously described using ImageJ software [17, 49].

### In vitro axon regeneration assay

Embryonic DRG tissue from E13.5 mice was dissociated in trypsin–EDTA (Thermo Fisher Scientific, 25300054) and cultured on a dish as previously described [12, 17]. The tissue culture plates were then coated with poly-d-lysine (Sigma, P0899) and laminin (Thermo Fisher Scientific, 23017015). The culture medium consisted of Neurobasal Medium (Thermo Fisher Scientific, 21103049) supplemented with 2% B-27 (Thermo Fisher Scientific, 17504044), 1% Glutamax (Thermo Fisher Scientific, 35050061), 1 μM 5-fluoro-2′-deoxyuridine (Sigma, F0503), 1 μM uridine (Sigma, U3003), 1% penicillin–streptomycin (Thermo Fisher Scientific, 15070063), and 50 ng/mL 2.5S nerve growth factor (Envigo, BT-5017). To knock down PDK1, the indicated lentivirus was added to the culture at DIV2. Cultured neurons were re-plated at DIV5 and then incubated at 37 °C in 5% CO_2_ for 5 min with DMEM (Hyclone, 500mlsh30243.01)/0.05% trypsin–EDTA mixture (1:1). Cells were dissociated using gently pipetting, plated to new culture plates, and incubated for 14 h at 37 °C in 5% CO_2_. The re-plated cells were fixed for 15 min in 4% paraformaldehyde and subjected to immunocytochemistry using anti-SCG10 and TUJ1 antibodies. Axonal lengths were measured using ImageJ. GSK2334470 (Selleckchem, S7087) was added in dissociated cells and mixed with pipetting.

### RNA extraction and quantitative RT-PCR

Total RNA was extracted from primarily cultured embryonic DRG cells using an RNAqueous-Micro Total RNA Isolation Kit (Ambion, AM1931) following the manufacturer’s instructions. DNase I (Thermo, EN0521) was added to the RNA sample for 20 min at 37 °C. The DNase reaction was terminated using DNase inactivation reagent (Invitrogen, 8174G) at room temperature for 2 min. The supernatant was then transferred to a NanoDrop spectrophotometer to determine RNA concentration. Quantitative RT-PCR was performed using PowerUP SYBR Green Master Mix (Thermo, A25918) following the kit manual.

## Data Availability

Not applicable.

## Abbreviations

Ax: Axotomy
CNS: Central nervous system
DRG: Dorsal root ganglion
KD: Knockdown
NS: Not significant
PNS: Peripheral nervous system
shRNA: Short hairpin RNA
